# Metabolic Effects of Berries with Structurally Diverse Anthocyanins

**DOI:** 10.3390/ijms18020422

**Published:** 2017-02-15

**Authors:** John Overall, Sierra A. Bonney, Mickey Wilson, Arnold Beermann, Mary H. Grace, Debora Esposito, Mary Ann Lila, Slavko Komarnytsky

**Affiliations:** 1 Plants for Human Health Institute, North Carolina State University, North Carolina Research Campus, 600 Laureate Way, Kannapolis, NC 28081, USA; jcoveral@ncsu.edu (J.O.); sabonney@ncsu.edu (S.A.B.); mlwilso8@ncsu.edu (M.W.); babeermann@davidson.edu (A.B.); mhgrace@ncsu.edu (M.H.G.); daesposi@ncsu.edu (D.E.); mlila@ncsu.edu (M.A.L.); 2 Department of Food, Bioprocessing & Nutrition Sciences, North Carolina State University, 400 Dan Allen Drive, Raleigh, NC 27695, USA; 3 Department of Animal Science, NC State University, 120 Broughton Drive, Raleigh, NC 27695, USA; 4 Department of Biology, Davidson College, 405 N Main St., Davidson, NC 28035, USA

**Keywords:** berry, gut microbiome, obesity, inflammation, functional food

## Abstract

Overconsumption of energy dense foods and sedentary lifestyle are considered as major causes of obesity-associated insulin resistance and abnormal glucose metabolism. Results from both cohort studies and randomized trials suggested that anthocyanins from berries may lower metabolic risks, however these reports are equivocal. The present study was designed to examine effects of six berries with structurally diverse anthocyanin profiles (normalized to 400 µg/g total anthocyanin content) on development of metabolic risk factors in the C57BL/6 mouse model of polygenic obesity. Diets supplemented with blackberry (mono-glycosylated cyanidins), black raspberry (acylated mono-glycosylated cyanidins), blackcurrant (mono- and di-glycosylated cyanidins and delphinidins), maqui berry (di-glycosylated delphinidins), Concord grape (acylated mono-glycosylated delphinidins and petunidins), and blueberry (mono-glycosylated delphinidins, malvidins, and petunidins) showed a prominent discrepancy between biological activities of delphinidin/malvidin-versus cyanidin-type anthocyanins that could be explained by differences in their structure and metabolism in the gut. Consumption of berries also resulted in a strong shift in the gastrointestinal bacterial communities towards obligate anaerobes that correlated with decrease in the gastrointestinal luminal oxygen and oxidative stress. Further work is needed to understand mechanisms that lead to nearly anoxic conditions in the gut lumens, including the relative contributions of host, diet and/or microbial oxidative activity, and their implication to human health.

## 1. Introduction

Obesity development and diabetes risks are intimately linked through alterations in insulin signaling and glucose metabolism [1]. The metabolic stressors are often underpinned by physiological changes which ultimately lead to the development of insulin resistance, hyperglycemia, and chronic low-grade inflammation in key metabolic as well as gastrointestinal tissues [2]. Previous studies in animal models as well as in humans indicated that dietary supplementation with anthocyanin-containing foods influenced many cardiovascular and metabolic outcomes [3]. These findings were supported by epidemiological observations [4] and randomized controlled trials using berries [5,6] or mixtures of partially purified anthocyanins [7]. However, not all results were consistent between the studies [8]. While berries with primary malvidins and delphinidins generally improved metabolic and cardiovascular disease risk biomarkers [6,9,10,11], cyanidins offered little or no protection in many cases [12,13,14]. Previously, we observed that blackcurrant delphinidins were also more likely to confer favorable metabolic adaptations compared to cyanidins in the diet-induced obese (DIO) mouse model [15]. Taken together, these findings raised the question of the major differences between various anthocyanins with respect to structure and associated bioactivity.

Anthocyanins are present in plants in different concentrations and with various degrees of hydroxylation, methylation, glycosylation, and acylation [16]. Gastrointestinal metabolism of the primary anthocyanins partially explained a previous perception of their extremely poor bioavailability after oral administration [17]. The majority of dietary anthocyanins were not absorbed at the upper gastrointestinal level and reached the intestinal microbiome that biotransformed them into phenolic metabolites [18]. Parent aglycone structures and secondary modifications were critical to their stability, bioavailability, and transformation in the gastrointestinal lumen. The in vivo conversion of hydroxylated anthocyanins to their respective methylated counterparts (i.e., malvidins) via Phase II methylation reactions was necessary for enhanced tissue uptake and bioactivity [19], while glycosylation and acylation were associated with increased stability and reduced bioactivity [20,21].

This important gap in knowledge on the effect of structural differences of the primary anthocyanins and their biological activities represents a major hindrance to achieving consensus over the efficacy and the most effective strategy of dietary supplementation with anthocyanin-rich foods to achieve favorable metabolic and cardiovascular outcomes. The present study was therefore designed to examine changes in metabolic risk factors after consumption of six berries with structurally diverse anthocyanin profiles. Primary anthocyanins were identified in feces of the DIO mice with intact and disrupted gut microbiome and correlated to several measures of metabolic health, including body composition, hyperglycemia, insulin resistance, microbiome profile, and gastrointestinal luminal oxygen and oxidative stress.

## 2. Results

### 2.1. Characterization of Whole Berry Anthocyanins

**Figure 1:**
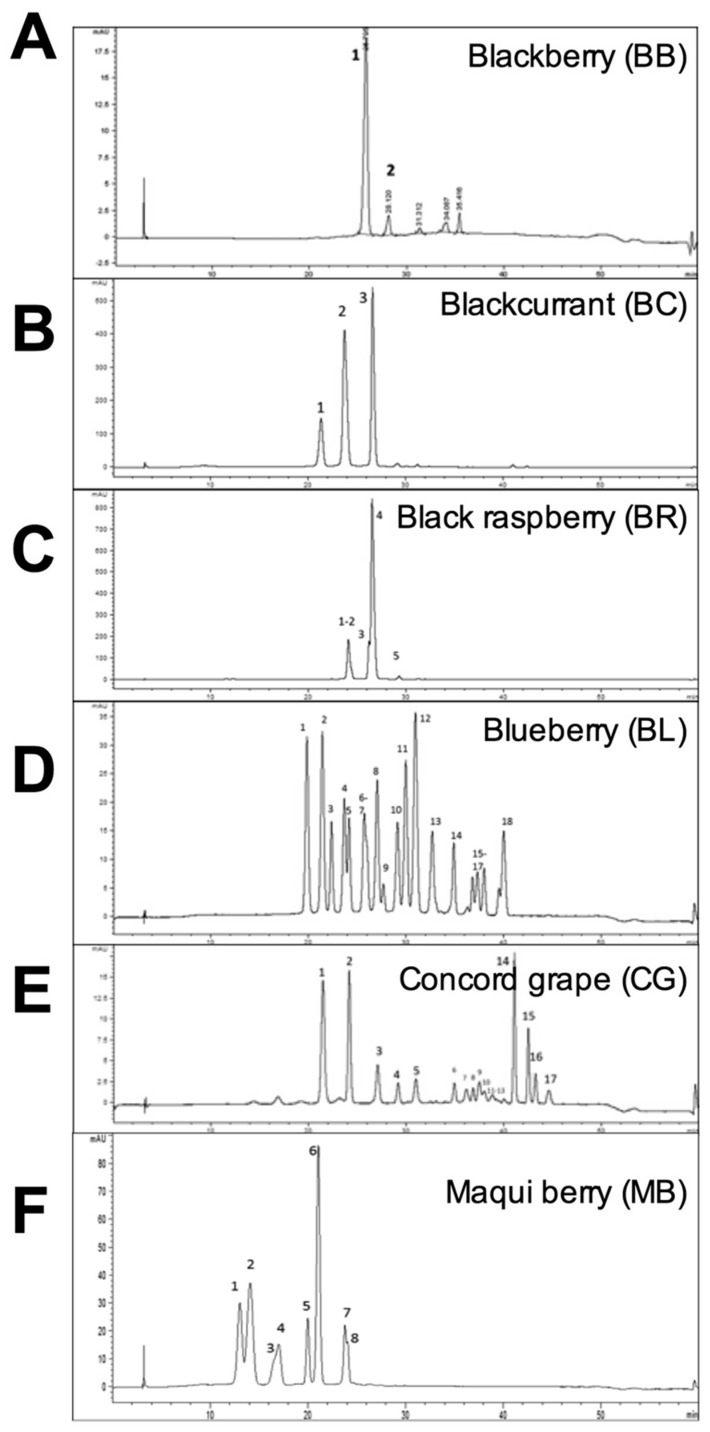
HPLC analysis of anthocyanins from whole freeze dried berries including (**A**) blackberry; (**B**) blackcurrant; (**C**) black raspberry; (**D**) blueberry; (**E**) Concord grape; and (**F**) maqui berry. Peak numbers correspond to Table 1 labels and order.

The HPLC-DAD analysis was used to characterize and quantify anthocyanins in freeze-dried powders from whole berries and fecal samples [22,23,24,25] (Figure 1). The primary anthocyanins in blackberry and black raspberry were monoglycosylated cyanidins (93%) and diglycosylated cyanidins (91%), respectively, in good agreement with previously reported anthocyanin profiles for these berries [26,27]. Blackcurrant and maqui berry contained delphinidins as primary (58% and 78%) and cyanidins as secondary (42% and 21%) anthocyanins, and most pigments were present in the diglycosylated, non-acylated form as reported earlier [28,29]. Blueberry and Concord grape had the most complex anthocyanin profiles enriched with malvidins (34% and 7%), petunidins (23% and 19%), and delphinidins (29% and 37%), which were predominantly monoglycosylated and non-acylated (blueberry) or acylated (Concord grape), as observed previously [30,31]. Blueberry (100%) and blackberry (93%) were the best sources of monoglycosylated anthocyanins, while blackcurrant (84%) and maqui berry (62%) were predominantly diglycosylated. Acylation was highest in anthocyanins from Concord grape (54%). Major anthocyanins quantified in all samples were listed in the order of elution in Table 1 and their compositional differences were summarized in Table 2. Freeze dried whole berry powders were then normalized to the total anthocyanin content and incorporated into the high fat diet (HFD) to contain 400 µg/g total anthocyanins from each berry. Anthocyanin levels in the diet remained stable for at least four days at room temperature and for the duration of the study when diets were stored at −80 °C.

### 2.2. Changes of Body Weight and Food Intake

**Figure 2:**
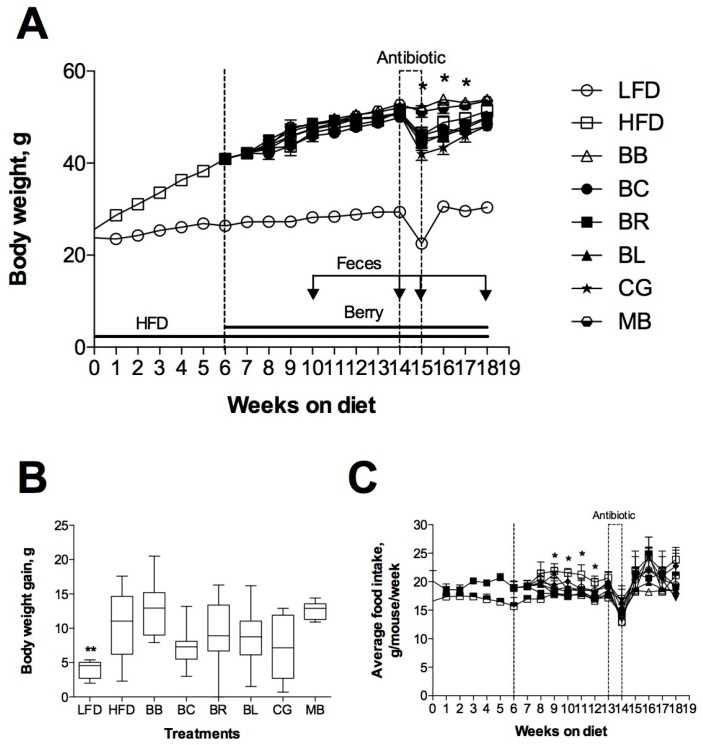
Effects of berry supplementation on: body weight (**A**); body weight gain (**B**); and food intake (**C**) of C57BL/6J mice. Mice were fed low fat diet (LFD) or high fat (HFD) for six weeks. DIO mice were further randomized to control (HFD) and berry diets (BB, blackberry; BC, blackcurrant; BR, black raspberry; BL, blueberry; CG, Concord grape; MB, maqui berry) normalized to contain 400 µg/g anthocyanins for additional 12 weeks. An antibiotic cocktail was administered for one week in drinking water (Weeks 14–15 of the study) and feces were collected as indicated by arrows. Results are expressed as means ± SEM, *n* = 8. Body weight and food intake were analyzed by 2-factor repeated measures ANOVA, with time and treatment as independent variables. * *p* < 0.05, ** *p* < 0.01 vs. HFD control.

Six-week-old mice were fed LFD or HFD for six weeks to initiate development of obesity in the HFD animals. Next, the HFD treatment groups were randomized to individual berry diets for an additional 12 weeks. There were no abnormal clinical signs throughout the entire study. After 18 weeks on HFD, obese mice developed 68.7% larger body weights as compared to their LFD controls (51.3 g versus 30.4 g, Figure 2A). Incorporation of berry powders into the diets for 12 weeks resulted in 6% smaller body weights in animals consuming blackcurrant or Concord grape, and a 3% decrease in body weight of animals treated with blueberry (not significant at *p* < 0.05). Body weight loss was absent in animals consuming blackberry, black raspberry, or maqui berry. Antibiotic cocktail given to animals in drinking water for one week to suppress gastrointestinal microbiome (Week 8–9 on berry diets) resulted in a prominent 10%–15% body weight loss in all LFD and HFD animals (*p* < 0.05).

This effect was significantly attenuated in animals consuming blackberry or maqui berry and correlated to the final body weight gains in these groups (Figure 2B). Antibiotic cocktail had a similar effect on food intake of all animals (Figure 2C), however all mice recovered their body weights and food intakes once antibiotics were removed from the drinking water. Consumption of Concord grape increased average food intake (13.7%, *p* < 0.05) and this effect was not observed in other treatments.

### 2.3. Changes in Body Composition

**Figure 3:**
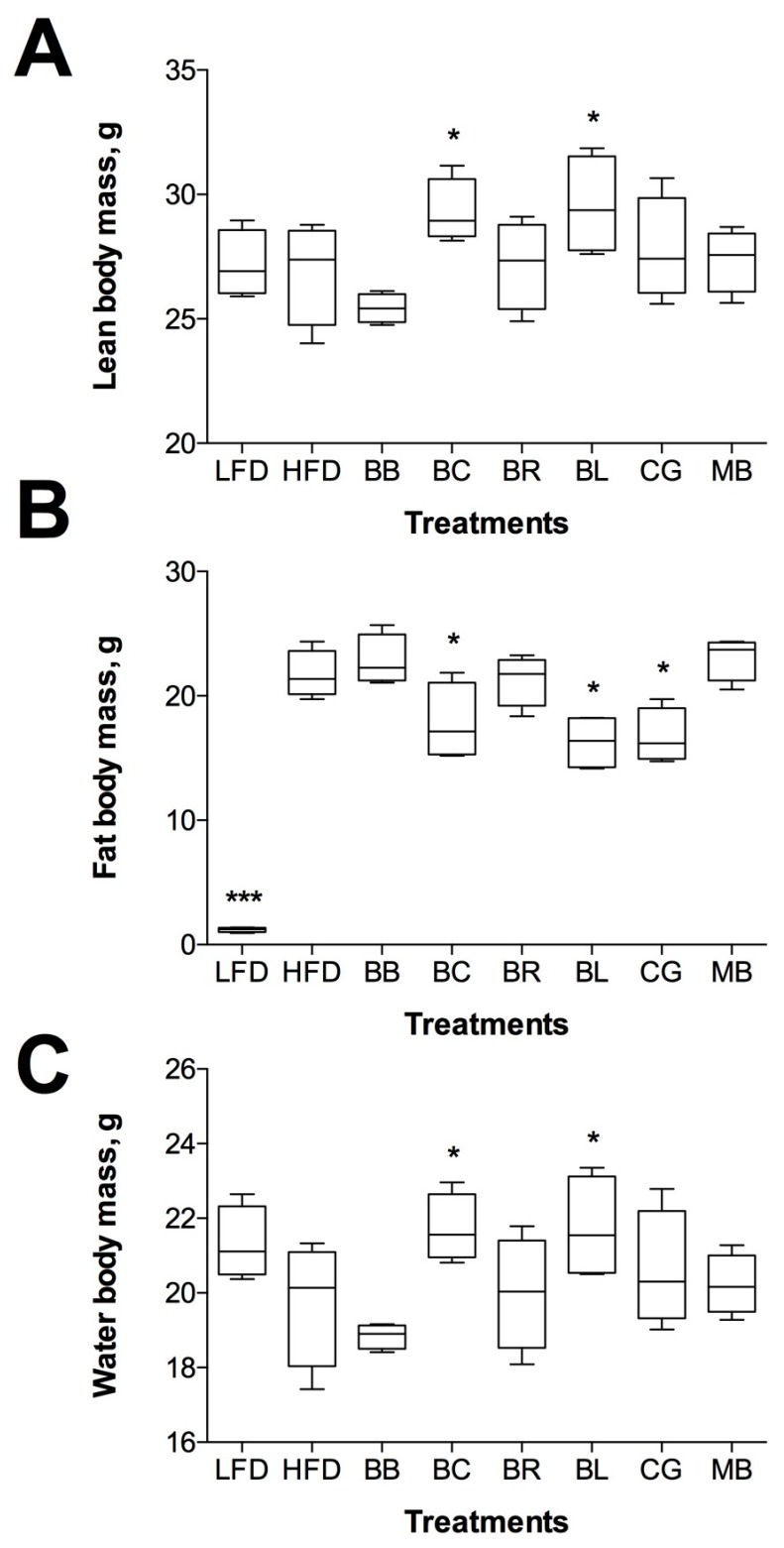
Effects of berry supplementation on body composition: (**A**) lean body mass; (**B**) fat body mass; and (**C**) total water are reported as means ± SEM. * *p* < 0.05 and *** *p* < 0.001 when compared to HFD by one-way ANOVA followed by Dunnett’s post hoc test.

Berry supplementation had differential effects on body composition of the treated animals. Consumption of blueberry and blackcurrant markedly increased lean body mass (9.8%–10.2%, *p* < 0.05), water mass (9.9%–10.1%, *p* < 0.05), and concurrently decreased fat body mass in these animals (−17.9%–24.6%, *p* < 0.05), resulting in a non-significant net decrease in body weight gain (Figure 3). Supplementation with Concord grape slightly decreased fat body mass, while black berry, black raspberry, and maqui berry supplementation had no significant effect on body composition.

### 2.4. Anthocyanins in Fecal Samples

When compared at different time points (4, 8, and 12 weeks on berry diets), anthocyanins were detectable in feces of all treatments groups, albeit at different amounts (Table 3). Feeding with blueberry and blackberry resulted in low anthocyanin content of the feces after four weeks of supplementation (2.37–5.30 μg/g), suggesting that most of the ingested parent anthocyanins were absorbed, broken down, and/or metabolized in the gastrointestinal tract. Blackcurrant and maqui berry showed higher resistance to gastrointestinal break down and accumulated in feces in 4–10-fold concentrations as compared to the other berries (26.35–44.93 μg/g). This trend persisted in Weeks 8 and 12 of the study. Addition of the antibiotic cocktail to the drinking water for one week visually intensified red/purple coloration of the feces collected during this time. Disruption of the gastrointestinal microbiome resulted in remarkable accumulation of the parent anthocyanins in feces, with maqui berry, blackcurrant, and Concord grape being most affected (260.58–339.32 μg/g). The enhanced accumulation of anthocyanins was detectable to a lesser degree in black raspberry (120.97 μg/g) and virtually undetectable in blackberry and blueberry (8.51–46.57 μg/g).

### 2.5. Effect on Glucose Metabolism and Insulin Sensitivity

**Figure 4:**
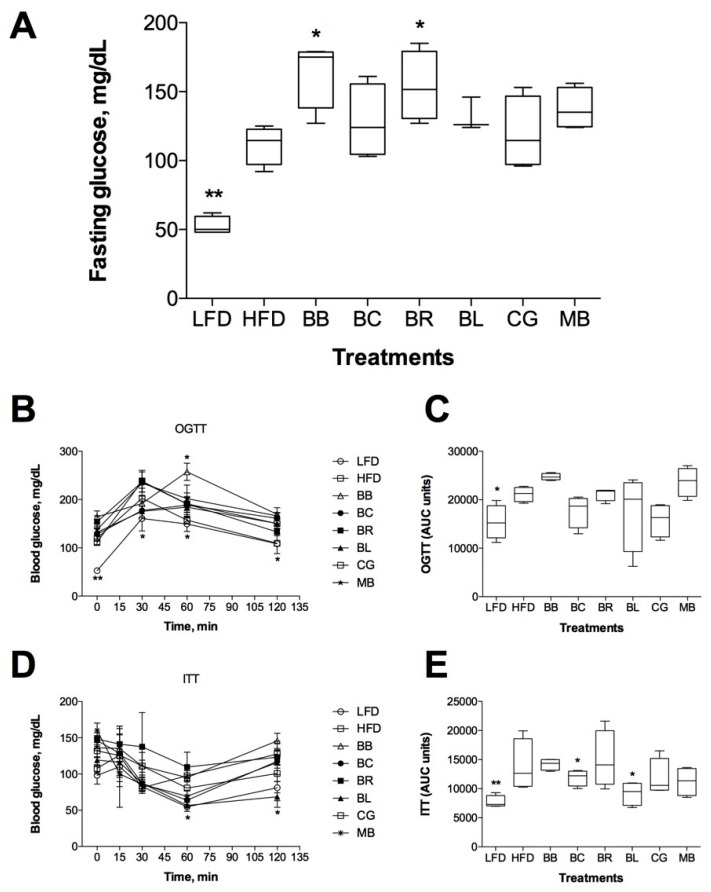
Insulin-sensitizing effect of berry supplementation: (**A**) Fasting blood glucose; (**B**) oral glucose tolerance test; and (**C**) respective AUCs; and (**D**) insulin tolerance test; and (**E**) respective AUCs are reported as means ± SEM. * *p* < 0.05 and ** *p* < 0.01 when compared by two-factor repeated measures ANOVA (**B**,**D**); or one-way ANOVA followed by Dunnett’s post hoc test.

Baseline blood glucose levels were measured on Week 12 of berry supplementation after overnight fast (Figure 4A). HFD animals showed significantly increased blood glucose levels as compared to LFD controls (136 vs. 65 mg/dL, *p* < 0.01). Supplementation with blackberry and black raspberry led to a modest increase in fasting glucose (153–164 mg/dL, *p* < 0.05), while the rest of the berries did not affect blood glucose levels. However, berry consumption directly affected glucose and insulin tolerance in these animals. While blood glucose levels of the HFD controls were significantly higher at 30, 60, and 120 min after oral glucose treatment as compared with the LFD mice, animals consuming blackcurrant, blueberry, and Concord grape showed a trend for lower peak glucose concentrations that did not reach significance (Figure 4B,C). These effects were more pronounced in the insulin tolerance test, when animals supplemented with black currants and blueberry showed increased insulin sensitivity similar to the LFD controls (Figure 4D,E).

### 2.6. Changes in Microbiome and Gastrointestinal Lumen Oxygen

**Figure 5:**
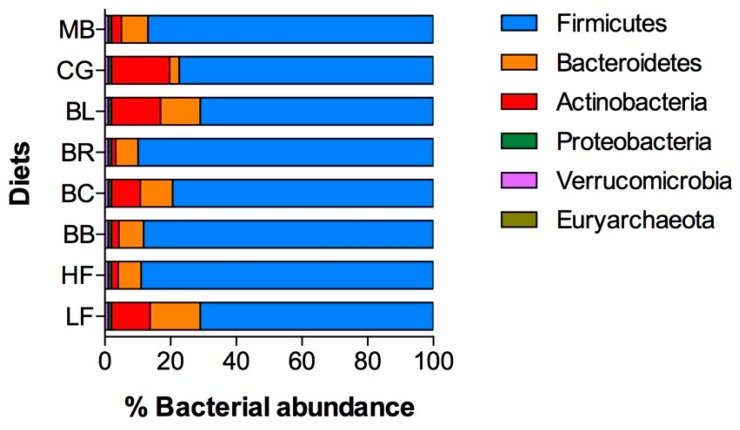
Changes in gut microbiota profiles following 12-week berry supplementation. Relative abundance of bacteria phyla in feces samples as measured by GULDA qPCR array indicated significant increase in obligate anaerobe populations of Bacteroidetes and Actinobacteria in berry-supplemented HFD animals (especially in blueberry and blackcurrant), similar to the LFD controls.

Gastrointestinal bacterial profiles were strongly affected by HFD and berry diets. Analysis of bacterial relative abundance in fecal samples showed that Firmicutes were the largest group of bacteria in healthy LFD animals (71%) that was further expanded to 89% with HFD diet. This expansion was achieved by the significant decreases in the obligate anaerobe populations of Bacteroidetes and Actinobacteria (Figure 5). Berry supplementation with blackberry and black raspberry did not change this trend. Animals consuming Concord grape showed the largest expansion of Actinobacteria from 2% to 18% relative to HFD controls. Blueberry and blackcurrant supplementation was associated with significant shifts in the fecal bacterial profiles that simultaneously increased populations of obligate anaerobes Bacteroidetes (from 7% to 10%–12%) and Actinobacteria (from 2% to 9%–15%). While the mechanism responsible for these shifts are not clear, it is possible that consumption of berries and specifically anthocyanins reduced oxygen tension in the gut lumens, thus promoting growth of oxygen-sensitive bacterial populations.

**Figure 6:**
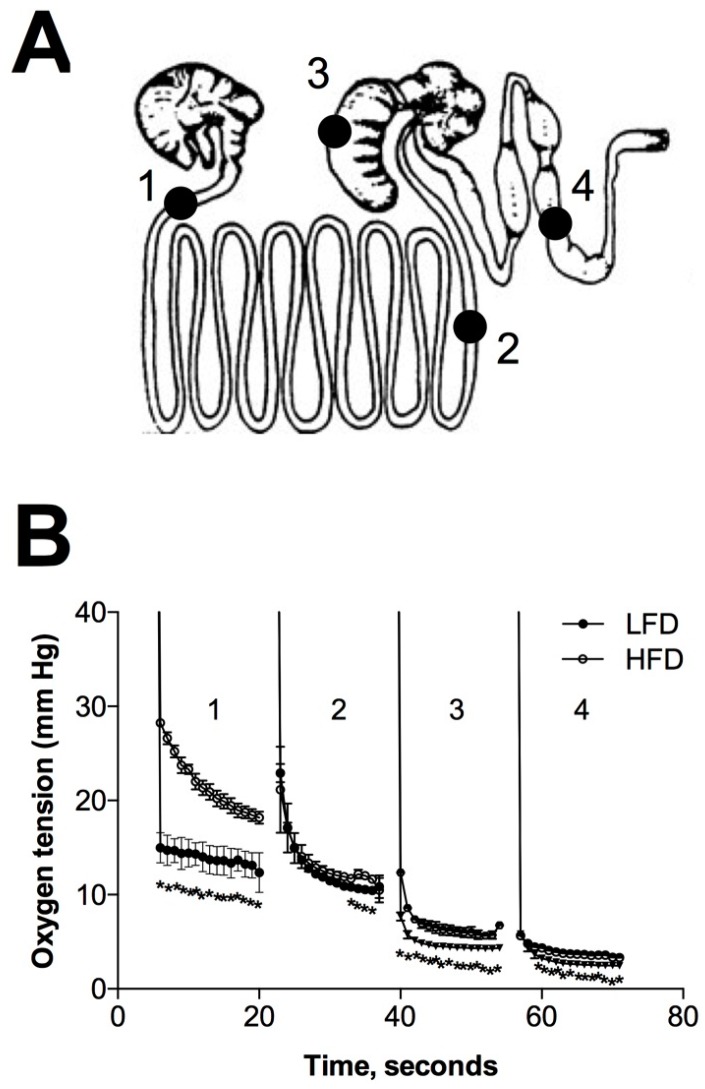

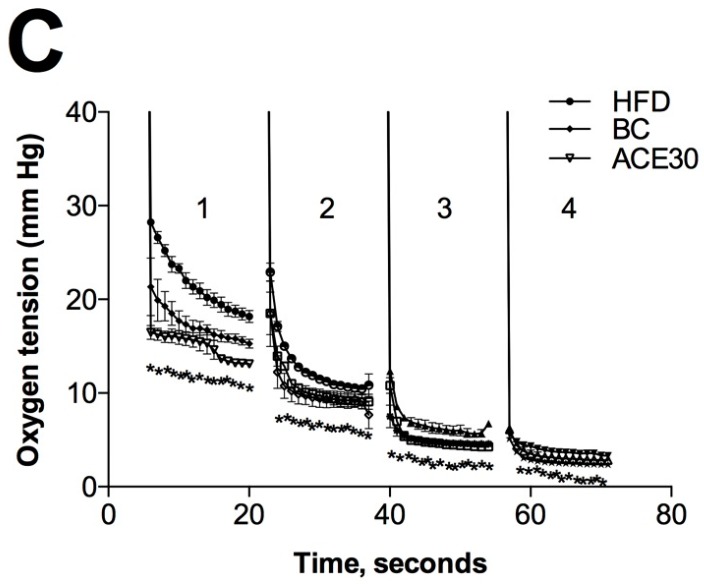
Gut luminal oxygenation profiles of different gastrointestinal regions of mice following 12-week berry supplementation: (**A**) schematic representation of mouse gastrointestinal tract including duodenum (1), ileum (2), cecum (3), and colon (4) sections; (**B**) oxygen tension curves of LFD and HFD animals indicated significant increases in gut luminal oxygen content of the HFD animals; and (**C**) oxygen tension curves in mice supplemented with blackcurrant or blackcurrant anthocyanins (ACE30 extract) showed decreased oxygen concentration within the gastrointestinal lumen when compared to HFD controls. Data are reported as means ± SEM. * *p* < 0.05 when compared to by two-factor repeated measures ANOVA.

To answer this question, we focused on the blackcurrant as this berry was highly enriched with anthocyanins and largely devoid of other polyphenols [32]. We used 0.3 mm Clark-type oxygen microelectrode to measure pO_2_ in the four sections of the gastrointestinal lumen including duodenum, ileum, cecum, and colon in animals fed LFD or HFD diet (Figure 6A). The oxygen tension gradually decreased along the gastrointestinal tract, suggesting that oxygen entered the gut during feeding and was depleted as the food moved posteriorly. HFD diet was associated with higher oxygen tension in all compartments of the gastrointestinal lumen (Figure 6B), with highest differences found in the duodenum (15 vs. 22 mmHg), cecum (4 vs. 6 mmHg) and colon (2.6 vs. 3.6 mmHg). Remarkably, blackcurrant diet reduced luminal pO_2_ values near to the LFD baseline in all four gastrointestinal compartments tested (Figure 6C). This effect could be largely attributed to the anthocyanins present in the blackcurrant, as it was more profound in animals fed HFD supplemented with 1% ACE30 anthocyanin-rich blackcurrant extract that was effective at reducing metabolic risk factors in DIO mice previously [15].

## 3. Discussion

A single serving of anthocyanin-rich berries may contribute in excess of 100–200 mg anthocyanins to a regular diet [33], which is 5–10-fold higher than the daily intake of other flavonoids [34]. However, anthocyanins from different plants show various degrees of hydroxylation, methylation, glycosylation, and acylation [16]. This may explain the equivocal results from animal and human studies, since predominantly delphinidin- and malvidin-containing fruits are more likely to improve metabolic and cardiovascular risk factors (i.e., blueberry [6], black currants and bilberry [9,10], or grapes [11]), while cyanidins offer less protection (i.e., elderberry [12], blood orange [13], or purple carrot [14]). 

To understand the relationship between structural constraints and biological activity of anthocyanins in the native food matrix, we selected six berries with diverse anthocyanin compositions for this study (Table 1). Blackberry and black raspberry both contained cyanidins as primary anthocyanins, yet with contrasting glycosylation profiles. Blackcurrant and maqui berry predominantly contained delphinidins with diglycosylation profile similar to black raspberry. Blueberry contained monoglycosylated delphinidins, malvidins, and petunidins (opposite of blackberry), while Concord grape was selected for high presence of acylated delphinidins, malvidins, and petunidins (Table 2). Despite diverse anthocyanin profiles, all berries contained sufficient amounts of total anthocyanins to allow for normalized incorporation of berries into HFD. The final diets contained from approximately 0.5% (black raspberry) to 5% (Concord grape) of the freeze-dried whole berry powders (*w*/*w*) and delivered 400 µg/g food of total anthocyanins. In this study, animals consumed an average of 2.85 ± 0.34 g food/mouse/day (71.3 mg/kg/day), thus ingesting 1.14 mg/mouse/day (28.5 mg/kg/day) of total anthocyanins. When translated to humans, this treatment was equivalent to consuming 2.4 mg/kg/day or 145 mg/day of total anthocyanins for an average adult [35] and could be easily achieved by daily consumption of 1–2 servings of fresh berries [33]. Normalization for anthocyanin content did not allow us to control the treatments for different sugar levels. All berries had similar carbohydrate profiles (10–17 g/100 g fresh weight (FW)), however Concord grapes contained more sugars (16 g/100 g FW) than the rest of the berries (4–10 g/100 g FW).

Dietary supplementation with monoglycosylated cyanidins (blackberry) and diglycosylated cyanidins (black raspberry) had no effect on body weight, food intake, body composition and metabolic risk factors (fasting blood glucose and insulin sensitivity) in the HFD mice (Figure 2, Figure 3 and Figure 4). Previously, whole powdered black raspberry supplementation did not prevent the development of obesity or improved lipid status in HFD mice [19] and it was suggested that complex glycosylation nature of black raspberry anthocyanins precluded their ability to modulate metabolic health [36]. A similar observation was made for complex acylated cyanidins from purple carrot [14], diglycosylated cyanidins from elderberry [12], acylated and diglycosylated cyanidins from blood orange [13], and monoglycosylated cyanidins from jaboticaba [37]. Weak antidiabetic effects were reported in animals for cyanidins from purple corn [38], black soybeans [39], purple sweet potato [40], mulberry [41], cherry [42], chokeberry [43] or highly purified cyanidin-3-glucoside alone [44]. Taken together, our data strongly suggested that cyanidin-based dietary interventions would be much less effective in alleviating metabolic risk factors than previously thought.

Primary diglycosylated delphinidins from black currants (non-acylated) and maqui berry showed similar effects on body weight, body composition, and measures of insulin resistance in the HFD animals. While blackcurrant showed higher efficacy at improving the metabolic outcomes associated with obesity and diabetes, supplementation with maqui berry at physiological levels of dietary intake had limited effect on these parameters, likely due to high proportion of di- and tri-glycosylated anthocyanins in the fruit. Previously, hypoglycemic effects of maqui berry were reported for supraphysiological levels of supplementation with the anthocyanin-enriched extract (100–500 mg/kg) and purified delphinidin 3-sambubioside-5-glucoside (425 mg/kg) [45]. Beneficial metabolic effects of black currant anthocyanins have been also described [15,46]. We observed a similar outcome from consumption of blueberry monoglycosylated delphinidins, malvidins, and petunidins (non-acylated) as compared to the Concord grape (acylated). Berries with acylated anthocyanin profiles showed less biological activity when consumed in physiological concentrations. Direct comparison between monoglycosylated anthocyanins from blueberry and diglycosylated anthocyanins from black currants suggested that supplementation with monoglycosylated molecules is more beneficial to the metabolic health (Figure 2, Figure 3 and Figure 4). There are two possible explanations for the observed differences between cyanidin- and delphinidin/malvidin/petunidin-type anthocyanins. It is very likely that anthocyanins with the free hydroxyl groups are less stable in the gastrointestinal environment and do not depend on gastrointestinal bacteria for their metabolism and absorption (delphinidin being an exception as it can be *O-*methylated to form petunidin or malvidin in vivo). This property was particular evident in animals with disrupted gut microbiome (Table 3). At the same time, *O-*methylated metabolites from malvidins and petunidins (including *O-*methyl delphinidins) were characterized with increased hydrophobicity at the B-ring of the molecule that reduced the plasma residence time and increased tissue affinity [47], thus being more biologically active. Complex glycosylation and acetylation patterns reduced bioactivity of anthocyanins, but greatly increased their stability in the gastrointestinal tract. When gut microbiome was disrupted with antibiotics, we observed 4–10-fold increase in diglycosylated and acylated anthocyanins excretes in feces. The effect was strongest in animals consuming maqui berry: dietary supplementation with 400 µg/g anthocyanins resulted in 339 µg/g anthocyanins excretion rate in feces upon suppression of gastrointestinal bacteria with the antibiotic cocktail (Table 3). The data suggested that subjects with gut microbiomes disrupted by a disease or lifestyle modification would have decreased benefits from dietary supplementation with berries as compared to their healthy counterparts, as described for black currants previously [15].

The gut microbiota in the LFD mice was dominated by Firmicutes (71%) and Bacteroidetes (15%), and the HDF diet increased this ratio to 89% and 7%, respectively, in strong agreement with previously reported analysis of the mouse gut metagenome [48]. We observed significant shifts in the gut microbiome profiles towards greater abundance of obligate anaerobes in animals supplemented with different berries, and these effects were most prominent in blackcurrant and blueberry groups (Figure 5). Since oxygen removal from the gut depends on food composition, microbial fermentation, and native oxidases secreted into the gut lumen [49], the changes in the oxygen content of the luminal environment may thereby modulate the composition of the gut microbiota. Indeed, consumption of HFD was associated with increased oxygen tension in all gut compartments, and this effect was reduced by incorporating berries and berry anthocyanins into the diet (Figure 6). Conversely, increase in host oxygenation altered luminal oxygenation in the gut and suppressed oxygen-intolerant bacterial populations [50]. The presence of marked hypoxia within the lumen of the distal parts of the gastrointestinal tract is therefore consistent with the known abundance of anaerobic bacteria at these sites, and our study indicates that gastrointestinal oxygenation can be modulated by dietary supplementation with berry anthocyanins.

## 4. Materials and Methods

### 4.1. Chemicals

The cyanidin-3-*O*-β-glucoside (HPLC grade standard) was purchased from Polyphenols Laboratories AS (Sandnes, Norway). All other chemical reagents and solvents were purchased from Sigma (St. Louis, MO, USA). Commercial whole freeze-dried berry powders were BB, blackberry (Nubeleaf, Portland, OR, USA); BC, blackcurrant (Just the Berries, Palmerston North, New Zealand); BR, black raspberry (BerriHealth, Corvallis, OR, USA); BL, blueberry (Wild Blueberry Association of North America, Old Town, ME, USA); MB, maqui berry (Sunburst Superfoods, Thornwood, NY, USA). Whole Concord grape puree was purchased from AgriAmerica (Silver Creek, NY, USA) and freeze-dried to obtain CG powder. A commercial blackcurrant powdered extract ACE30 (Active Cassis Extract 30) was kindly provided by Eddie Shiojima (Just the Berries, Palmerston North, New Zealand).

### 4.2. Animals and Diets

All animal experiments were approved by the North Carolina Research Campus Institutional Animal Care and Use Committee (IACUC protocols 12-018 and 16-011) in the David H. Murdock Research Institute, the AAALAC accredited animal care facility.

Male, 6-week-old C57BL/6J mice were purchased from the Jackson Laboratory (Bar Harbor, ME, USA) and housed four animals per cage under controlled temperature (24 ± 2 °C) and light (12 h light–dark cycle, lights on at 7:00 a.m.). Immediately upon arrival, animals were allowed to adapt to new conditions for 7 days and handling the animals was performed daily during this time to reduce the stress of physical manipulation. Mice were then randomized into ad libitum access to Research Diets (New Brunwick, NJ, USA) low 10 kcal % fat diet D12450J (low fat diet, LFD, 3.85 kcal/g, *n* = 12) or high 60 kcal % fat diet D12492 (high fat diet, HFD, 5.24 kcal/g, *n* = 56) and tap water for 6 weeks. Obese mice were further randomized to control high fat diet (HFD, *n* = 8) or berry-supplemented treatment groups normalized to 400 µg/g total anthocyanins (berry powders incorporated into HFD by Research Diets). Mice were kept on the respective diets for an additional 12 weeks. Animal weight and food intake (accounting for spillage) were recorded weekly for the duration of the study. All animal diets were kept at −80 °C for long-term storage and stability, and freshly thawed food was dispensed to animals every 3–4 days to limit phytochemical degradation in food matrix. Body composition analysis was performed on unanesthetized mice using EchoMRI (Echo Medical Systems, Houston, TX, USA) during Week 1 and Week 12 of the study.

### 4.3. Antibiotic Knockdown of Endogenous Gut Microbiome

An antibiotic cocktail (0.5 g/L vancomycin, 1 g/L neomycin sulfate, 1 g/L metronidazole, 1 g/L ampicillin) previously shown to be sufficient to deplete all detectable commensal bacteria [22] was administered in drinking water ad libitum to all animals for 1 week (between Week 8 and 9 of HFD treatment).

### 4.4. Oral Glucose and Insulin Tolerance Tests

For oral glucose tolerance test, mice were fasted overnight (16 h) and received oral gavage of d-glucose (1.5 g/kg body weight). For insulin tolerance test, mice were fasted for 4 h and received intraperitoneal injection of insulin (0.75 U/kg body weight, Santa Cruz Biotechnology, Santa Cruz, CA, USA). Blood glucose concentrations were measured at 0, 15, 30, 60 and 120 min after glucose or insulin challenge in blood samples obtained from tail-tip bleedings, using a glucometer (Lifescan, Johnson and Johnson, New Brunswick, NJ, USA).

### 4.5. Sample Collection and Oxygen Measurements

At the end of the experiment, mice were euthanized and blood was collected by heart puncture. Oxygen levels in freshly dissected digestive tracts were measured with 0.3 mm Clark-type oxygen microelectrode ET1125, EPU354 isoPod, and Pod-Vu software v5.5.20 (Edaq, Colarado Springs, CO, USA). Electrode was zeroed in water sparged with nitrogen (1 min/mL water) and calibrated in distilled water saturated with air (20.9% oxygen). The accuracy of the microelectrode to reach zero and 20.9% oxygen was rechecked periodically during experiments. Microelectrode was inserted through a small hole cut into the gut wall and all readings were completed in 15 s intervals within 4 min after dissection. Percent oxygen was measured in the duodenum, ileum, cecum, and colon at ambient temperature (23 ± 1 °C) and local barometric pressure (National Weather Service). Oxygen partial pressure (1 mmHg = 133.322 Pa = 1 torr) was calculated from percent oxygen reading recorded at −800 mV polarization.

Liver, fat, muscle, gastrointestinal tissues (stomach, duodenum, ileum, cecum and colon) and luminal digesta were collected and stored at −80 °C to determine the temporal sequence and signaling events that are responsible for changes in physiology and metabolism. Fecal samples from mice were collected, weighed, and pooled by cage at four time points, including Week 4 (1 month on HFD), Week 8 (2 months on HFD, start of antibiotic treatment), Week 9 (end of antibiotic treatment), and Week 12 (3 months on HFD) and stored at −80 °C.

### 4.6. Gastrointestinal Microbial Profiles

Genomic DNA was extracted from mouse fecal samples using QIAamp Fast DNA Stool Mini kits (Qiagen, Germantown, MD, USA), quantified using Take3 plate and Synergy H1 microplate spectrophotometer (BioTek, Sunnyvale, CA, USA), and adjusted to 1 ng/µL. Quantitative real-time PCR was performed on an ABI 7500 Fast (Life Technologies, Carlsbad, CA, USA) in a total volume of 20 µL containing 10 µL 2× SYBR Green PCR Master Mix, 1 µL of each primer from GUt low-density array (GULDA) Array [23], 4.4 µL of nuclease-free water and 3.6 µL of template DNA. The amplification program consisted of 50 °C for 2 min; 95 °C for 10 min; 40 cycles of 95 °C for 15 s and 60 °C for 1 min; and a dissociation curve (95 °C for 15 s, 60 °C for 15 s, then increasing to 95 °C at 2% rate). The mean *C*t-value was determined based on a set threshold value of 0.2 and using the automatic baseline correction. Differences in *C*_t_-values for each bacterial target (N_0_-normalization) were calculated between those obtained with the universal and target-specific primers and log-transformed. Fold-changes for target amplicons were calculated as the (log 2) ratio of normalized abundances at different time points.

### 4.7. Anthocyanin Extraction and Quantification

Anthocyanins were extracted from berry powders and feces using 60% aqueous methanol (1% trifluoroacetic acid) following a previously reported method for rapid analysis of various anthocyanins in rats [24]. Total anthocyanins were determined using the pH differential method [25] by producing a rapid and reversible structural change (color shift) and quantifying the difference in absorbance at 520 nm using Synergy H1 (BioTek, Sunnyvale, CA, USA).

### 4.8. HPLC Analysis of Anthocyanins

Individual anthocyanins in berry powders and feces were quantified by HPLC as described in our previous publication [26]. Briefly, filtered samples were injected (10 μL) into a 1200 HPLC system (Agilent Technologies, Santa Clara, CA, USA) equipped with a UV–vis diode array detector (DAD), controlled-temperature autosampler (4 °C), and column compartment (30 °C) using a reversed-phase Supelcosil LC-18 column, 25 mm × 4.6 mm × 5 μm (Supelco, Bellefonte, PA, USA). Standard curves were calculated using peak areas at UV of 520 nm as cyaniding-3-*O*-glucoside equivalents.

### 4.9. Statistics

Data were analyzed by one-way ANOVA followed by Dunnett’s multiple-range tests using Prism 6.0 (GraphPad Software, San Diego, CA, USA). Body weight gain, glucose and insulin tolerance were analyzed by two-factor repeated-measures ANOVA, with time and treatment as independent variables. All data were presented as means ± SEM. Significant differences were accepted when the *p*-value was <0.05.

## 5. Conclusions

In conclusion, the data presented in this study agreed with the emerging findings from preclinical and human studies demonstrating that delphinidin- and malvidin-based anthocyanins, at concentrations attainable in human tissues, were more effective at improving key metabolic risk factors than their cyanidin-based counterparts. Complex glycosylation and acetylation patterns reduced bioactivity of anthocyanins, but greatly increased their stability in the gastrointestinal tract. Finally, diets supplemented with berry or anthocyanins reduced gut luminal oxygenation and promoted abundance of obligate anaerobic bacteria from Bacteroidetes and Actinobacteria phyla at these sites. Further work is needed to understand mechanisms that lead to nearly anoxic conditions in the gut lumens, including the relative contributions of host, diet and/or microbial oxidative activity, and their implication to human health.

## Figures and Tables

**Table 1:** Anthocyanin profiles of whole freeze-dried berry powders. Total anthocyanins were determined by the pH differential [25], while individual peaks were quantified by HPLC [26].

Berry	Anthocyanins (Total, mg/g Dry Weight)	Individual Anthocyanins	Percent % (Total Anthocyanins)
Blackberry (BB)	9.42 ± 0.03	1. Cyanidin-3-*O*-glucoside	77.58
		2. Cyanidin-3-*O*-rutinoside	6.90
		3. Non-identified	trace
		4. Cyanidin-3-*O*-(malonyl)glucoside	15.52
		5. Cyanidin-3-*O*-(dioxalyl)glucoside	trace
Black currant (BC)	16.42 ± 0.24	1. Delphinidin-3-*O*-glucoside	14.61
		2. Delphinidin-3-*O*-rutinoside	43.92
		3. Cyanidin-3-*O*-rutinoside	39.54
		4. Cyanidin-3-*O*-glucoside	2.01
Black raspberry (BR)	24.75 ± 1.19	1. Cyanidin-3-*O*-sambubioside	8.61
		2. Cyanidin-3-*O*-glucoside	8.62
		3. Cyanidin-3-*O*-(xylosyl)rutinoside	73.62
		4. Cyanidin-3-*O*-rutinoside	8.18
		5. Pelargonidin-3-*O*-rutinoside	1.13
Blueberry (BL)	9.33 ± 0.26	1. Delphinidin-3-*O*-galactoside	9.21
		2. Delphinidin-3-*O*-glucoside	10.22
		3. Cyanidin-3-*O*-galactoside	4.12
		4. Delphinidin-3-*O*-arabinoside	6.03
		5. Cyanidin-3-*O*-glucoside	4.34
		6. Petunidin-3-*O*-galactoside	7.72
		7. Cyanidin-3-*O*-arabinoside	2.63
		8. Petunidin-3-*O*-glucoside	7.51
		9. Peonidin-3-*O*-galactoside	1.44
		10. Petunidin-3-*O*-arabinoside	5.33
		11. Malvidin-3-*O*-galactoside	8.81
		12. Malvidin-3-*O*-glucoside	12.70
		13. Malvidin-3-*O*-arabinoside	5.12
		14. Delphinidin-3-*O*-(acetyl)glucoside	3.54
		15. Cyanidin-3-*O*-(acetyl)glucoside	1.43
		16. Malvidin-3-*O*-(acetyl)galactoside	2.11
		17. Petunidin-3-*O*-(acetyl)glucoside	2.24
		18. Malvidin-3-*O*-(acetyl)glucoside	4.83
Concord grape (CG)	2.37 ± 0.03	1. Delphinidin-3-*O*-glucoside	13.61
		2. Cyanidin-3-*O*-glucoside	12.01
		3. Petunidin-3-*O*-glucoside	7.41
		4. Peonidin-3-*O*-glucoside	5.92
		5. Malvidin-3-*O*-glucoside	6.42
		6. Delphinidin-3-*O*-(acetyl)glucoside	5.75
		7. Delphinidin-3,5-*O*-(coumaroyl)diglucoside	5.60
		8. Cyanidin-3-*O*-(acetyl)glucoside	5.31
		9. Cyanidin-3,5-*O*-(coumaroyl)diglucoside	6.55
		10. Petunidin-3-*O*-(acetyl)glucoside	trace
		11. Malvidin-3,5-*O*-(coumaroyl)diglucoside	trace
		12. Peonidin-3,5-*O*-(coumaroyl)diglucoside	trace
		13. Peonidin-3-*O*-(acetyl)glucoside	trace
		14. Delphinidin-3,5-*O*-(coumaroyl)diglucoside	11.36
		15. Cyanidin-3-*O*-(coumaroyl)glucoside	8.05
		16. Petunidin-3-*O*-(coumaroyl)glucoside	6.24
		17. Peonidin-3-*O*-(coumaroyl)glucoside	5.77
		18. Malvidin-3-*O*-(coumaroyl)glucoside	trace
Maqui berry (MB)	10.95 ± 0.12	1. Delphinidin-3-*O*-sambubioside-5-*O*-glucoside	15.36
		2. Delphinidin-3,5-*O*-diglucoside	23.91
		3. Cyanidin-3,5-*O*-diglucoside	trace
		4. Cyanidin-3-*O*-sambubioside-5-*O*-glucoside	11.32
		5. Delphinidin-3-*O*-sambubioside	7.76
		6. Delphinidin-3-*O*-glucoside	31.26
		7. Cyanidin-3-*O*-glucoside	6.86
		8. Cyanidin-3-*O*-sambubioside	3.53

**Table 2:** Summary of anthocyanin diversity in whole freeze-dried berry powders (percent of total).

Berry	Anthocyanidins (Aglycones)					Glycosylation Ratio (Di/Mono)	Acetylation Ratio (Yes/No)
	Cy	De	Mv	Pt	Peo		
Blackberry (BB)	100	0	0	0	0	7/93	16/84
Black currant (BC)	42	58	0	0	0	84/16	0/100
Black raspberry (BR)	99	0	0	0	0	91/9	0/100
Blueberry (BL)	12	29	34	23	2	0/100	13/87
Concord grape (CG)	32	37	7	19	7	23/77	54/46
Maqui berry (MB)	21	78	0	0	0	62/38	0/100

**Table 3:** Total anthocyanins recovered from feces (µg/g DW).

Diet *	Time on Diet, Weeks			
	4	8	9 (1 Week Antibiotic)	12
HFD	nd	nd	nd	nd
Blackberry (BB)	2.37 ± 0.08	3.48 ± 0.10	8.51 ± 0.18	4.83 ± 1.13
Black currant (BC)	26.35 ± 1.25	34.26 ± 0.14	326.82 ± 31.89	48.17 ± 2.74
Black raspberry (BR)	44.93 ± 6.64	62.10 ± 9.42	120.97 ± 2.13	83.93 ± 1.30
Blueberry (BL)	5.30 ± 0.66	17.05 ± 3.10	46.57 ± 6.00	18.57 ± 0.91
Concord grape (CG)	9.24 ± 5.13	22.41 ± 3.74	260.58 ± 10.46	34.26 ± 0.60
Maqui berry (MB)	30.38 ± 1.18	31.22 ± 1.37	339.32 ± 52.69	172.16 ± 5.44

* Diets were normalized to 400 µg/g total anthocyanins (0.04%) by incorporating 0.5%–5% of whole freeze-dried berries; nd, not detected.
